# Development and validation of climate and ecosystem-based early malaria epidemic prediction models in East Africa

**DOI:** 10.1186/1475-2875-13-329

**Published:** 2014-08-22

**Authors:** Andrew K Githeko, Laban Ogallo, Martha Lemnge, Michael Okia, Ednah N Ototo

**Affiliations:** Kenya Medical Research Institute, Centre for Global Health Research, (KEMRI) Climate and Human Health Research Unit, PO Box 1578, Kisumu, Kenya; IGAD Climate Prediction and Applications Centre (ICPAC), PO Box 10304-00100, Nairobi, Kenya; National Institute for Medical Research, NIMR Tanga Centre, Tanga, Tanzania; Uganda Ministry of Health, PO Box 7272, Kampala, Uganda

**Keywords:** Malaria, Epidemic prediction, Models, Africa

## Abstract

**Background:**

Malaria epidemics remain a serious threat to human populations living in the highlands of East Africa where transmission is unstable and climate sensitive. An existing early malaria epidemic prediction model required further development, validations and automation before its wide use and application in the region. The model has a lead-time of two to four months between the detection of the epidemic signal and the evolution of the epidemic. The validated models would be of great use in the early detection and prevention of malaria epidemics.

**Methods:**

Confirmed inpatient malaria data were collected from eight sites in Kenya, Tanzania and Uganda for the period 1995-2009. Temperature and rainfall data for the period 1960-2009 were collected from meteorological stations closest to the source of the malaria data. Process-based models were constructed for computing the risk of an epidemic in two general highland ecosystems using temperature and rainfall data. The sensitivity, specificity and positive predictive power were used to validate the models.

**Results:**

Depending on the availability and quality of the malaria and meteorological data, the models indicated good functionality at all sites. Only two sites in Kenya had data that met the criteria for the full validation of the models. The additive model was found most suited for the poorly drained U-shaped valley ecosystems while the multiplicative model was most suited for the well-drained V-shaped valley ecosystem. The +18°C model was adaptable to any of the ecosystems and was designed for conditions where climatology data were not available. The additive model scored 100% for sensitivity, specificity and positive predictive power. The multiplicative model had a sensitivity of 75% specificity of 99% and a positive predictive power of 86%.

**Conclusions:**

The additive and multiplicative models were validated and were shown to be robust and with high climate-based, early epidemic predictive power. They are designed for use in the common, well- and poorly drained valley ecosystems in the highlands of East Africa.

**Electronic supplementary material:**

The online version of this article (doi:10.1186/1475-2875-13-329) contains supplementary material, which is available to authorized users.

## Background

Malaria remains the most serious public health problem in Africa [1]. The disease transmission intensity has a spatial-temporal variation that is closely related to climate and weather. While climate refers to a mean state, the weather variability oscillates around the mean climate state. In the highlands of East Africa, climate and weather are major drivers of malaria transmission [2]. The major malaria transmission rate controlling factor in the East African highlands is temperature [3], however topography controls drainage and vector breeding and this can have significant effects on transmission and disease prevalence [4]. Highlands are defined as areas 1,500 m above sea level where the mean annual temperature used to be in the range of 16-19°C but have however become warmer due to climate change [5–7]. Below 18°C malaria transmission cannot take place because the malaria parasite *Plasmodium falciparum* takes 56 days to develop in *Anopheles gambiae,* whose mean life span is only 23 days [8]: thus, the vector will die before the parasite can mature and be transmitted. Climate change and variability can cause local temperatures to shift above the 18°C threshold creating suitable malaria transmission conditions. Events such as the 1997–8 El Niño that caused severe epidemics in the East African highlands was associated with 4°C anomalies in the mean monthly maximum temperatures [9], which would have driven the 18°C mean temperature to 22°C. Concurrent with heavy rainfall, this event caused server malaria epidemics in the East African highlands [10].

About 124.7 million people in Africa lived in malaria epidemic-prone areas in 2001 [11]. In Tanzania, 25% of the population lives in malaria epidemic-prone areas [12] and a similar proportion of the population in Kenya is at risk. Malaria epidemics are associated with high morbidity and mortality. Records spanning from 1990–97 in the western Kenya highlands indicate malaria accounted for 32% of hospitalized patients [13]. During an epidemic in western Kenya in the early 1990s, morbidity increased 3.7-fold and mortality 8.6-fold [14].

There has been a great need to develop a model for early prediction of malaria epidemics to enable early launching of interventions to prevent the health crises and disasters associated with epidemics [15, 16]. A weather-driven model was developed for predicting malaria epidemic in the highlands of western Kenya with a lead-time of two to four months between prediction and the occurrence of the epidemic [9]. This model provides for the first time an opportunity to prevent epidemics instead of attempting to manage them. The model is based on the identification of weather conditions that support rapid development of vectors in permanent breeding sites, an accelerated sporogonic cycle and the expansion of vector breeding habitats, conditions that lead to the evolution of an epidemic. The model uses mean monthly rainfall thresholds above which vector populations increase. The model explicitly assumes that malaria transmission can only occur in areas where the mean annual temperature is >18°C. It was determined that for an epidemic to occur a mean monthly temperature anomaly was first observed and then followed by rainfall above a specific threshold.

For the model to be used as an operational tool it required testing in different ecosystems using mean monthly, microscopically confirmed, malaria cases and temperature and rainfall data collected over a period of about ten years. During the testing period the models would be modified to function in specific ecosystems. Epidemiological and entomological research had indicated that despite similar rainfall and temperature in the western Kenya highlands there was an 8.5-fold difference in *P. falciparum* malaria prevalence [17] and three-fold difference in the abundance of *An. gambiae sensu lato* (*s.l.*) [18] between the U-shaped and the V-shaped valley ecosystems. This difference was attributed to the drainage qualities and the availability and stability of breeding habitats in the two ecosystems. The plateau ecosystem was found to behave like the V-shaped valley ecosystem.

In addition to fine-tuning the models for specific ecosystems, their sensitivity, specificity and positive predictive power were determined to confirm their reliability and robustness.

## Methods

### Study sites

The study sites were areas in Kenya, Tanzania and Uganda situated at 1,550 m above sea level with a history of malaria epidemics since the 1980s. The sites were required to have a hospital with microscopically diagnosed inpatient data and a meteorological station with monthly and rainfall data spanning 1960 to 2010. It was expected that four hospitals would be identified in each country. The hospitals selected are shown in Table 1 and the meteorological stations in Table 2.

**Table 1:** **The hospitals selected for malaria data collection**

Kenya	lat/lon/alt	Tanzania	lat/lon/alt	Uganda	lat/lon/alt
**St Marys Mukumu Hospital**	0.214233 34.766922 1,600 m	Rubya Hospital	-1.333753 31.809626 1,500 m	Kabale District Hospital	-1.24685 29.986839 1,800 m
**Kisii District Hospital**	-0.679077 34.773102 1,814 m	Dareda Hospital	-4.240045 35.49232 1,600 m	Rukingiri District Hospital	-0.789532 29.925299 1,640 m
**Nandi South District Hospital**	0.134582 35.101318	Muleba Hospital	-1.837663 31.656933 1,500 m	Kisoro District Hospital	-1.281946 29.680853 1,900 m
**Litein Hospital Kericho**	-0.581278 35.19011 1,900 m			Nyakibale District Hospital	-0.795968 29.929676 1,600 m

**Table 2:** **Meteorological stations where temperature and rainfall data were collected**

Kenya	Tanzania	Uganda
Kakamega	Arusha	Kabale
Kericho	Bukoba	Mbarara
Kisii		
Eldoret		

### Malaria and meteorological data collection

At the beginning of malaria data collection from hospitals, it was realized that the distribution of insecticide-impregnated bed nets had started in some sites in 2005 and malaria infections had started declining due to this intervention. In other sites, some indoor residual spraying had been carried out intermittently and this too had affected malaria transmission. This was more common in Kenya. It was then decided that data from 1995–2004 only should be used for the model development and testing. In other sites in Uganda and Tanzania data were available from 2000–2010 and the intervention histories were not well known. Once collected, the data were tested for seasonality as this is an indicator of consistency with changes in rainfall and temperature. Lack of seasonality is an indicator of low quality in data recording.

Meteorological data, including monthly rainfall, maximum and minimum temperatures, were obtained from Departments of Meteorology in Kenya, Tanzania and Uganda. The data were checked for completeness and duration of collections. In the case of southwestern Uganda, data were only available from the Kabale station and temperature data were missing for several years. Although data were available from Mbarara airport, the station was too far from the hospital where malaria data were collected and, therefore, was unsuitable for analysis. In Tanzania, data from the Bukoba station were used to model malaria data from Muleba as there was no station in the highland site. In this case, while the absolute temperatures between Muleba and Bukoba are different, it is expected that their anomalies would be similar and they could be used as input into the Muleba model.

### Mosquitoes breeding and topography

The flat area in a valley bottom with a slope close to zero is an indicator of the availability of larval breeding habitat, the size of the adult population and the level of malaria transmission. Historical and georeferenced larval distribution data in the western Kenya highlands were extracted from Climate and Human Health Research databases. The area occupied by larvae and the slope were estimated. The angles of inclination of the rivers were calculated. The ratio of the surface with less than 4°C and the slope of the river were used as an indicator of the risk of malaria transmission in a valley ecosystem. These ratios were used as guide of distinguishing the U- and V-shaped valleys systems.

### Model construction

The model construction was carried out as described previously [9]. The sequences of weather events that lead to the evolution of an epidemic are anomalous temperatures followed by rainfall that exceed certain thresholds that are ecosystem specific. In poorly drained valley ecosystems, the threshold is 150 mm/month and in the well-drained valley ecosystems, the threshold is 250–300 mm/month. Generally epidemics are triggered by temperature that are >2°C above normal for a given month that precede the rains. There is usually a lag of one month between the occurrence of anomalous temperatures and precipitation above the entomological rainfall thresholds. The epidemic occurs one or two month after the beginning of the rains.

The model consists of logical statements that are designed to filter biologically significant signals from temperature and rainfall data with a lag between the temperature and rainfall events. The strength of the temperature/rainfall composite index is then expressed as an epidemic risk. In the U-shaped valley there is usually a lag of two months between anomalous temperatures and a malaria epidemics but a lag of three months has been observed if the rains are prolonged. High rainfall and high temperature anomalies increases the risk of an epidemic. In addition, prolonged rainfall increases the size of the epidemic. In the V-shaped valley, there is a lag of three months between the anomalous temperatures and the epidemic.

### Data transformation and noise reduction

The rainfall data are transformed from continuous variable into discreet code numbers to reduce the noise from the real signal. The malaria mean monthly inpatient numbers are expressed as departure from a long-term mean of all the cases for all the months under observation. The long-term mean malaria cases are calculated as follows;


Per cent monthly departure of malaria cases from the long-term mean;


Where,

PDLTM = Per cent departure from long-term mean

*M*_*cases*_ = monthly number of malaria cases.

### Models

There are two general models referred to and these are the additive and the multiplicative models. In the additive models, the temperature anomaly codes and rainfall codes are added together and in the multiplicative model the parameters are multiplied. Temperature and rainfall data were transformed from continuous to discrete variables referred to as codes. The additive model can have variants. In some cases the temperature anomaly codes respond to exponential functions and in other cases they respond to simple arithmetic codes. The additive model best fits the poorly drained U-shaped valley ecosystems and is more responsive to temperature anomalies than rainfall. Another variant of the model is the +18°C model that uses 18°C as the threshold temperature for the evolution of an epidemic. This model does not need temperature climatology data to calculate monthly anomalies.

The contribution of the mean temperature anomalies, from the mean monthly maximum and minimum temperature varies between different sites. For example, in Kakamega, western Kenya all the temperature anomalies originate from the mean monthly maximum temperature, whereas in Arusha, Tanzania, 90% of the anomalies originate from the minimum temperature. In the case of Kakamega the model only responds to maximum temperatures.

The multiplicative model variants arise from different rainfall thresholds and these are site specific. It was found that in this system several incidents of anomalous temperature occur with no subsequent rainfall. This results in false signals. The problem is solved by multiplying the temperature and rainfall codes so that when the rainfall code is zero, then the product of the terms is also zero. This model works best in the well-drained, V-shaped valleys, which require heavy rainfall to trigger significant changes in mosquito populations.

### The additive model



### The additive model with exponential temperature effect



### Multiplicative model: Nandi



### Additive +18°C model



Where,

T^*i*^ = temperature code at month *i*

R^*i+2*^ = Rainfall code at month *i + 2*

T^*m*^ = highest temperature anomaly code in the climatology data

R^*m*^ = maximum rainfall anomaly code in the climatology data

ER^*i+4*^ = epidemic risk at month *i + 4*

Data transformation is indicated in Additional file 1.

### Testing the model performance

Specificity, sensitivity and positive predictive power of the models were carried out as previously described [19]. Only models that met the testing criteria were tested. The criteria required that the hospitals and corresponding meteorological station should be at similar altitude and that the distance between the two should less that 60 km. Furthermore, high-quality hospital and meteorological data must be available. Only data from two sites in Kenya met these criteria: Kakamega and Nandi districts. The Kakamega site is largely dominated by U-shaped valleys while Nandi is dominated by V-shaped valleys. Ten-year malaria data from each of the two sites (1995–2004) were available for the model validation.

### Model automation

The models were programmed in Microsoft Excel spreadsheet to allow simple entry of monthly temperature and rainfall data to compute the anomalies and plot the results in a graph.

## Results

### Epidemic threshold

An epidemic was defined as a 100% or a twofold or greater increase in confirmed malaria cases above the long term mean. Increases below this threshold were defined as seasonal outbreaks. In general, medical facilities are overwhelmed by number of cases during an epidemic and this can lead to high mortality.

### Epidemic risk threshold (ERT)

The epidemic risk value is the output of the model and it is ecosystem and site specific. Its value is determined by the temperature, rainfall and drainage efficiency of the ecosystem. The critical epidemic threshold value corresponds to 100% increase in malaria cases above the long-term mean. In general, the hotter and wetter it is, the higher is the epidemic risk. In the case of the additive Kakamega model, the ERT was 30% while for the multiplicative model in Nandi, it was it was 20%.

### Types of models tested

Three types of models were tested for functionality at eight sites (Table 3). There were three additive models and four multiplicative and one +18°C model. These are shown on Table 3.

**Table 3:** **The type of model developed at each site**

Site	Country	Valley Type	Model type
Kakamega	Kenya	U Shaped	Additive
Nandi	Kenya	V shaped	Multiplicative
Kericho	Kenya	V shaped	Multiplicative
Kisii	Kenya	V shaped	Multiplicative
Rubya Bukoba	Tanzania	U Shaped	Additive
Arusha Dareda	Tanzania	U Shaped	Additive
Muleba	Tanzania	V shaped	Multiplicative
Nyakibale model	Uganda	U shaped	+18°C model

### Additive model output: Kakamega

**Figure 1:**
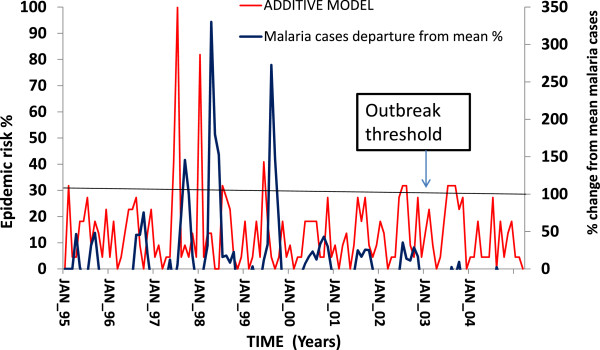
**The additive model output and the trends in malaria cases at Kakamega from 1995–2004.**

The additive model preformed very well using the Kakamega meteorological data (Figure 1). The model correctly predicted all epidemics and there were no false negatives or positives (Figure 1). Of the 122 events in the model, four were true epidemics (Table 4). This model has a low rainfall threshold of 150 mean monthly rainfall. The model responded to an exponential value of the maximum temperature anomaly (T^2^).

**Figure 2:**
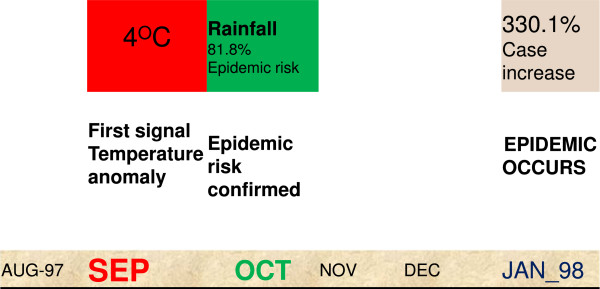
**The sequence of events starting with the observation of anomalous mean monthly temperature, followed by rainfall and finally the epidemic.**

Kakamega is a U-shaped ecosystem with poor drainage allowing for accumulation of water and formation of larval breeding habitats. The sequence of meteorological events and the evolution of an epidemic are shown on Figure 2. During the 1997–8 El Niño event an anomaly in the mean maximum monthly temperature of 4°C was observed in September 1997. Very heavy rainfall followed in October 1997 and the model indicated an epidemic risk of January 1998 that resulted in a 330.1% increase in malaria cases (Figure 2).

**Table 4:** **The sensitivity, specificity and positive predictive power of the additive model at Kakamega**

U-shaped ecosystem model performance, Kakamega			
**Event**	**Epidemic positive**	**Epidemic negative**	**Total**
**Model Positive**	*True positive (TP)*	*False positive (FP)*	
	4	0	4
**Model negative**	*False negative (FN)*	*True negative (TN)*	
	0	118	118
**Total**	4		122
**Sensitivity**	TP/(TP + FN)		1
**Specificity**	TN/(TN + FP)		1
**Positive predictive power**	TP/(TP + FP)		1

### Multiplicative data output: Nandi South

**Figure 3:**
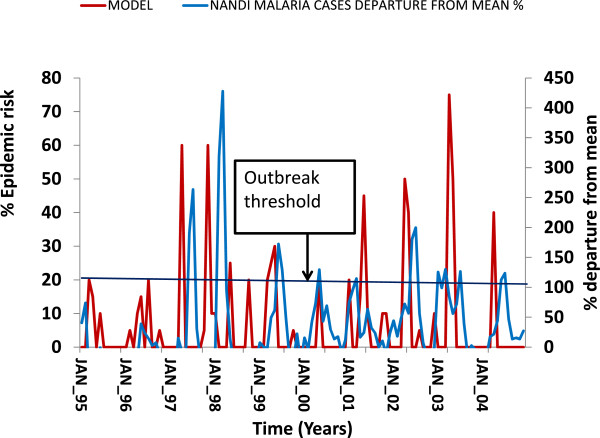
**The multiplicative model output and the trends in malaria cases at Nandi from 1995–2004.**

In the multiplicative model using data from Kericho meteorological station and malaria data from Nandi South Hospital, 118 events were recorded (Table 5, Figure 3). Of these, six were true positives, two were false negatives and one was a false negative. The sensitivity was 0.75 specificity 0.99 and positive predictive power 0.86 (Table 5). This model had a rainfall threshold of 200 mm mean monthly rainfall and responded to a linear temperature change. The variability in the maximum temperature contributed 89% of the anomalies and 11% from the minimum temperature. Nandi South district is a V-shaped ecosystem with good drainage that does not support good and permanent larval breeding habitats (Figure 3).

**Table 5:** **The sensitivity, specificity and positive predictive power of the multiplicative model at Nandi**

V-shaped ecosystem model performance, Nandi district			
**Event**	**Epidemic positive**	**Epidemic negative**	**Total**
**Model positive**	*True positive (TP)*	*False positive*	7
	6	1	
**Model negative**	*False negative (FN)*	*True negative (TN)*	111
	2	109	
**Total**	8	110	118
**Sensitivity**	TP/(TP + FN)		0.75
**Specificity**	TN/(TN + FP)		0.99
**Positive predictive power**	TP/(TP + FP)		0.86

### +18°C model

This model was developed for ecosystems without suitable climatology data. The recommended climatology data is from 1960–1990, a period that had no malaria epidemics in the East African highlands. The model can assume the multiplicative or the additive forms. A multiplicative form of the model was tested against a climatology-based model developed for Muleba district in northwest Tanzania. The +18°C model was able to explain 80% of the variation of the climatology-based model prediction. The Muleba model was not tested for its positive predictive power because at some point between 1997–2008 interventions using insecticide-impregnated bed nets were implemented and the exact period is unknown. However, the model had excellent performance in 1997–2003, which is most likely the pre-intervention period. Insecticide-impregnated nets suppress malaria epidemics. During this period the +18°C and the additive models epidemic predictions were in total agreement.

### Valley shapes and drainage

**Figure 4:**
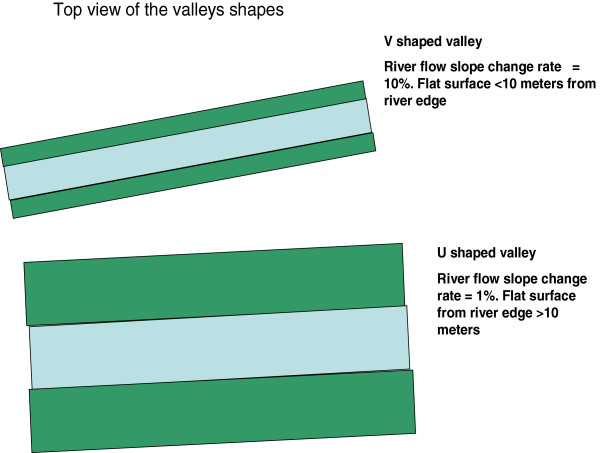
**The difference in the layout of -U- and V-shaped valleys.**

Analysis of terrain characteristics and mosquito breeding habitats resulted in the development of two rules for defining the U- and V-shaped valleys systems. Figure 4 shows the rules for determining the shapes of the valleys and thus the drainage classification of a particular ecosystem. The threshold rainfall is determined by plotting rainfall against malaria cases and determining the rainfall threshold that causes an epidemic. The blue area in Figure 4 represents the river and the green area the flat surface in the valley bottom.

### Model



Where risk factor is a variable proportional to mosquito productivity; distance represents the perpendicular distance with slope <4° from the river edge to the valley edge; slope = slope of the river in degrees. The terrain characteristics affect drainage of precipitation and control the rainfall thresholds for the epidemic prediction model (Figure 4).

## Discussion

While malaria is a major health burden in Africa, epidemics have increased their threat to human populations in sub-Saharan Africa. A major strategy for the control of an epidemic is the early detection of its evolution [20, 21]. This has led to a concerted effort by the international community to develop such systems [22, 23]. Since the late 1980s an increasing frequency of malaria epidemics was observed in the East African highlands and they often caught the health authorities by surprise, leaving them little time to respond. Despite the application of the WHO framework for malaria early warning systems [24], the lead-time between epidemic detection and response remained, at best, two weeks. Clearly this lead-time did not provide sufficient time for an effective response, particularly in remote villages and during the heavy rain season.

Githeko and Ndegwa [9] developed a climate-based, early epidemic prediction model that had to a lead-time of two to four months between the detection of the climate epidemic signal and the occurrence of the epidemic. While the model provided new potential for a true early epidemic warning system, it required wide testing in a variety of eco-epidemiological situations in the East African highlands. At the time of the development of the first models it was not known how the ecosystems affected the models’ performance or how the different levels of immunity would impact the sensitivity of the models.

The first step to further development and testing of the model was collecting data of laboratory-confirmed malaria cases, temperature and rainfall from several sites in the highlands of Kenya, Tanzania and Uganda. The major goal was to collect data spanning at least ten years in at least four sites in the highlands of each collaborating country. Two major challenges were encountered, these being incomplete datasets and the launch of malaria control interventions using insecticide-impregnated bed nets and indoor residual spraying. In Kenya, while data were available from 1995 it was known that malaria control interventions started in 2005 and thereafter malaria case trends did not fully respond to seasonal climate changes. In the case of Kenya, data from 1995–2004 were used for developing and testing new models. Another challenge was the distance between the health facility and the nearest meteorological station. While a meteorological station can predict rainfall and temperature within a radius of 60 km, many stations in Tanzania and Uganda were much further than this. In Uganda there was a problem of missing meteorological and malaria cases data. As a result of these challenges data from only two sites in Kenya, Kakamega and Nandi, were suitable for sensitivity, specificity and positive predictive power testing.

Initially it was envisaged that models would be site specific, in other words, different models for different sites, however it became clear that the models were responding to two ecosystems. Parasitological, immunological [17] and entomological data [18] indicated that that there were three ecosystems in the highlands that were defined by the drainage quality. The U-shaped valleys have a broad bottom with slow-flowing water allowing extensive breeding of mosquitoes, high and stable malaria transmission and a high frequency of immune response to malaria antigens. In contrast the V-shaped valley had narrow bottom, fast-flowing water and few stable vector-breeding sites. Consequently malaria transmission is unstable, and there was a low frequency of immune response to malaria antigens. The plateau transmission characteristics were similar to those of the V-shaped valley. More specifically, while the prevalence of *P. falciparum* in schoolchildren was 22.6% in the U-shaped valley ecosystems, during the period of the development of the models, it was 2.6% in the V-shaped valleys ecosystems. The prevalence of circumsporozoite protein (CSP) and merozoite surface protein (MSP) antibodies was 23.2 and 8.8% in the U-shaped and V-shaped ecosystems, respectively [17]. Vector abundance was three-fold greater in the U-shaped valleys compared to the V-shaped valleys [18].

The additive model had the best predictions for the U-shaped valley ecosystems while the multiplicative model had the best predictions for the V-shaped ecosystems. Both models had high positive predictive power. The high sensitivity and specificity indicated that the chance of an error was very small. In the case of the additive model in Kakamega, all the epidemic events were correctly identified. In the Nandi model there was only one false positive. The +18°C model performed as well as the additive model using the Muleba data in Tanzania.

The first signal in the model was anomalous mean, monthly temperature of >2°C before the beginning of the rainy season. If the mean monthly rainfall that followed this event exceeded the threshold, then an epidemic would occur one or two months later. This time lag provided sufficient time to launch interventions and prevent or minimize the impact of the epidemic.

End users of the models in the health and meteorological sectors from Kenya, Tanzania and Uganda were trained on how to construct the models and use them. In order to simplify the use of the models they were programmed in Microsoft Excel. The input into the model was mean monthly temperature and rainfall data and the output was a graph showing the epidemic risk. The risk was the final confirmation that an epidemic would occur one to two months later.

The model can also be used to simulate epidemics. During the seasonal climate, outlook forums of the Greater Horn of Africa region, a regional rainfall forecast, is provided indicating if the rainfall will be below, normal or above normal. Estimates of the rainfall and temperature using analogous years have been used as inputs in the models to predict the likelihood of a malaria epidemic. The models have been accurate in identifying seasonal risks of malaria epidemics. The forecast are made at least one month before the onset of the rainy season. El Nińo events can also be used to simulate epidemic risks by using data from analogous years to predict epidemics.

Most of the epidemics identified in the retrospective data from 1995 occurred during El Nino events in the East African highlands. It should be noted that data obtained during the ongoing malaria control programmes were unsuitable for model construction and the malaria cases do not resonate with the seasonal climate changes. While malaria cases have declined and epidemics seem to have disappeared, the climate risk may be increasing due to climate change and variability. It is, therefore, advisable that continuous surveillance of climate risks and intervention efficacy are carried out.

Epidemic malaria remains a threat in Kenya, Tanzania, Uganda, Ethiopia, Rwanda, and Burundi. There is a need to adopt these models and fine-tune them so that they can be used in all affected highlands in these countries. While it has been known that rainfall and temperature are major drivers of malaria epidemics [12, 15, 23, 25, 26] the role of topography, drainage and immunity has not been factored into the development of early epidemic prediction models in the past.

## Conclusions

This paper indicates that besides the temperature and rainfall variability, the evolution of malaria epidemics is significantly influenced by the topography and drainage quality of the ecosystems. The tested models exhibited high sensitivity, specificity and positive prediction values and with a lead-time between the climatic signals and malaria epidemics of two to four months. This lead-time provides an early warning to enable launching of epidemic interventions in order to prevent potential health disasters. The automation of the models provides a user-friendly tool for health and meteorological personnel involved in health-disaster prevention.

## Electronic supplementary material

Additional file 1: **Coding methodology (examples).** (DOC 26 KB)
