# Supplementary material for: Development and validation of climate and ecosystem-based early malaria epidemic prediction models in East Africa
Source: Malar J. 2014 Aug 22;13:329. doi: 10.1186/1475-2875-13-329 (PMC4158077; doi:10.1186/1475-2875-13-329)
Supplement: Supplementary file 1 — Additional file 1: Coding methodology (examples). (DOC 26 KB) [file 12936_2014_3373_MOESM1_ESM.doc]

**Coding methodology (examples)**

Data transformation and coding was achieved by using rainfall and temperature baselines and then converted. For the rainfall data, bins of 50mm were created. The first bin with rainfall data below the threshold e.g. 150mm for the U shaped valley was given a value of zero and thereafter each increment of 50mm was given a value of one. Temperature anomalies were calculated by subtracting the current month’s temperature from the long term mean temperature of that month. Bins were created where temperature anomalies less than 1°C were given the value of zero and each 1°C increase was given a value of one. For example, 1-1.9°C was transformed to 1, 2-2.9°C was transformed to 2, etc. This process converted continuous data to categorical data, a process that reduces background noise. For further details see Githeko and Ndegwa, 2001.

**Temperature**

**Programming Excel using logic filters for data transformation**

Programming Microsoft Excel™ spreadsheet to process the rainfall and temperature data requires the use of the *f_x_* IF (function). This function conducts conditional tests on values and formulas. The function statement includes a logic statement, a value if true and a value if false. For example:

**IF (logical_test, value_if_true, value_if_false)**

Formula with the IF function

1 logical test: The condition that you want to check.

2 value_if_true: The value to return if the condition is true.

3 value_if_false: The value to return if the condition is false.

The function looks at data in a particular location in the spreadsheet and returns the true and false values. Several statements can be constructed to carry out complex analysis of data. The spreadsheet was programmed to use serial logic tests and allocate values to temperature anomalies and rainfall discrete values (codes). The function also filters rainfall and temperature data values that do not contribute to the evolution of an epidemic. At the same time the function also transforms the data from continuous variables into discrete values thus reducing ‘noise’ from the original data.

**Exponential coding**

Cell A1 contains the temperature anomaly data. This formula contains four logic tests that return ***x^2^*** for true values as:

=IF(A1<1,1,IF(A1<2,4,IF(A1<3,9,IF(A1<4,16,25))))

**Arithmetic coding**

=IF(A1<1,1,IF(A1<2,2,IF(A1<3,3,IF(A1<4,4,5))))

**Rainfall coding**

The formula evaluates the rainfall data using a specific threshold, and allocates discreet values proportional to increasing rainfall. Poor drainage ecosystems (U-shaped valleys); threshold = 150 mm/month. Data with values below the threshold are allocated a value of zero.

Cell B1contains the mean monthly rainfall data

=IF(B1<**150**,0,IF(B1<175,1,IF(B1<200,2,IF(B1<225,3,IF(B1<250,4,5)))))

Well-drained ecosystem (V shaped valleys); threshold = 250 mm/month

=IF(B1<**250**,0,IF(B1<300,1,IF(B1<350,3,IF(B1<400,4,5))))
